# NK-cell dysfunction of acute myeloid leukemia in relation to the renin–angiotensin system and neurotransmitter genes

**DOI:** 10.1515/med-2022-0551

**Published:** 2022-09-20

**Authors:** Seyhan Turk, Ayriana Safari Baesmat, Aysegul Yılmaz, Can Turk, Umit Yavuz Malkan, Gulberk Ucar, Ibrahim Celalettin Haznedaroğlu

**Affiliations:** Department of Biochemistry, Faculty of Pharmacy, Hacettepe University, Ankara, 06105, Turkey; Department of Medical Microbiology, Faculty of Medicine, Lokman Hekim University, Ankara, 06105, Turkey; Department of Internal Medicine, Faculty of Science, Hacettepe University, Ankara, 06105, Turkey

**Keywords:** leukemia, natural killer cells, dysfunction

## Abstract

Acute myeloid leukemia (AML) is the most heterogeneous hematological disorder and blast cells need to fight against immune system. Natural killer (NK) cells can elicit fast anti-tumor responses in response to surface receptors of tumor cells. NK-cell activity is often impaired in the disease, and there is a risk of insufficient tumor suppression and progression. The aim of this study is to assess the dysfunction of NK cells in AML patients via focusing on two important pathways. We obtained single-cell RNA-sequencing data from NK cells obtained from healthy donors and AML patients. The data were used to perform a wide variety of approaches, including DESeq2 (version 3.9), limma (version 3.26.8) power differential expression analyses, hierarchical clustering, gene set enrichment, and pathway analysis. ATP6AP2, LNPEP, PREP, IGF2R, CTSA, and THOP1 genes were found to be related to the renin–angiotensin system (RAS) family, while DPP3, GLRA3, CRCP, CHRNA5, CHRNE, and CHRNB1 genes were associated with the neurotransmitter pathways. The determined genes are expressed within different patterns in the AML and healthy groups. The relevant molecular pathways and clusters of genes were identified, as well. The cross-talks of NK-cell dysfunction in relation to the RAS and neurotransmitters seem to be important in the genesis of AML.

## 1 Introduction

Acute myeloid leukemia (AML) is a phenotypically and genetically heterogeneous malignancy with an extremely poor prognosis due to disease recurrence being the major reason for therapy failure [1,2]. Comprehensive immunological profiling of newly diagnosed patients with AML reveals that those abnormalities in T-cell and natural killer (NK)-cell activity are the primary contributors to immune dysfunction, although B-cell function remains unaltered. Immunosuppression that leads to anti-leukemia immunity evasion is caused by T-cell maturity and depletion, as well as increased NK maturation and reduced T-cell activity. Effective therapeutic response to chemotherapy is associated with the restoration of T and NK function, and specific immunological markers are associated with overall survival [3,4].

Blood pressure, vascular resistance, and fluid/electrolyte balance are controlled by the renin–angiotensin system (RAS), which is a complex set of proteins that regulate these parameters. Also, RAS pathway genes have been identified in tissues, such as the bone marrow, where they have been demonstrated to be involved in leukemic hematopoiesis, or the generation of new blood cells. Studies have found that exposure to RAS inhibitors (RASi) can suppress the development of cancers via multimodal mechanisms and has attracted increased attention in the recent past. Due to their ability to limit tumor growth, proliferation, and metastasis, RASi are thought to be promising options for enhancing the effectiveness of chemoradiotherapy and targeted therapy [5].

Because of the variability and intricacy of the tumor microenvironment, AML remains untreatable. To develop successful treatment methods, it is critical to clarify the etiology of AML and to discover biomarkers related to leukemia. NK cells are a kind of cytotoxic immune cell that is capable of promptly recognizing and eliminating cancer cells. Analyzing tumor cells’ expression and kinds of NK-associated molecules might help predict how well patients will fare after being diagnosed with acute lymphoblastic leukemia (ND-AML).

A growing amount of research indicates that NK cells may be very effective against CML, AML, and MDS. But autoimmune condition processes generally prevent endogenous NK cells from performing their functions properly, resulting in poor tumor management and an increased risk of disease progression. While allogeneic NK cells have been shown to prevent leukemia recurrence in some circumstances, including stem cell transplantation, this therapy is not suitable for all patients. In addition, adoptively infused NK-cell-induced remissions are temporary and need repeated treatment to sustain persistent responses. As a result, novel techniques for inducing complete and lasting anti-leukemia responses in individuals with myeloid malignancies are required [6].

On the other hand, in recent years, scientists have focused increasing emphasis on traditional neurotransmitter receptors to get a deeper knowledge of the biology of leukemia and the cellular response to it [7]. The pharmacological suppression of dopamine and serotonin receptors, which both affect AML viability in relevant preclinical models, is used as a prognostic, diagnostic, and therapeutic target in AML [8]. Inhibition of both neurotransmitter receptors caused AML cells to undergo terminal differentiation, which had a significant impact on the leukemic cells at the very beginning of their differentiation process [9]. Differentiation-based treatments, which are more susceptible to chemo and lose their ability to self-renew, are appealing since they are frequent in all AML subtypes [10]. NK dysfunction has been seen in many hematological malignancies, including AML. However, in many of the AML cases, it is observed that NK cells cannot perform their anticancer functions appropriately. This might be related either to the number or dysfunction of NK cells.

The aim of the work was to compare the whole-genome data of NK cells isolated from AML patients and healthy individuals, to determine the genes, gene sets, and pathways responsible for the dysfunction of NK cells. In this study, we identified significant alterations in the expression of RAS and neurotransmitter genes in NK cells from AML patients and demonstrated how these changes could influence the anti-tumor capabilities of NK cells.

## 2 Methods

### 2.1 Obtaining and normalizing whole-genome expression of NK cells from healthy and AML patients

The Gene expression omnibus (GSE159624) was used to obtain data on human NK cells. The single-cell RNA profiling data of human bone marrow NK cells from healthy donors and AML patients were generated using the Illumina NextSeq 500 platform. The data were normalized in R (version 3.6.3) with the DESeq2 (version 3.9) package. The DESeq2 (version 3.9) package was designed for the purpose of normalizing, visualizing, and performing differential analysis on high-dimensional count data. To estimate log fold change and dispersion priors, and to construct posterior estimates for these values, it uses empirical Bayes methods [11]. Each sample has over 4,000 single cells with data on whole-genome expression. The average expression levels for each gene in the samples were determined.

### 2.2 Identification of RAS and neurotransmitter families related genes with differential expression in NK cells in healthy and AML patients

Using the limma (version 3.26.8) powers differential expression analysis, the whole normalized gene expression data of NK cells from healthy and AML patients were compared to identify the significance and differentially expressed RAS family and neurotransmitter pathway-related genes. Limma (version 3.26.8) is an R/Bioconductor (version 3.6.3) software tool for evaluating data from gene expression studies. It has a multitude of options for managing complicated experimental designs and borrowing information to overcome the issue of small sample numbers [12].

### 2.3 Hierarchical cluster analysis

A hierarchical clustering analysis was carried out to assess if the discovered genes could differentiate between the healthy and AML groups. Differentially expressed RAS genes and neurotransmitter pathways were grouped hierarchically using the similarity metric parameter and the Euclidean distance Gene Cluster v3.0 software as a full link.

### 2.4 Gene set enrichment analysis (GSEA)

GSEA was performed in accordance with the GSEA guideline method (http://software.broadinstitute.org/gsea/docGSEAUserGuideFrame.html). The study was conducted using GSE159624 data.

### 2.5 Pathway analysis

The “Database for Annotation, Visualization, and Integrated Discovery” software was used to deduce the biological relationships between these differentially expressed genes. We identified the functional pathways underlying the 100 most significantly differentially expressed genes between the healthy and AML groups.

## 3 Results

### 3.1 RAS and neurotransmitter gene families differentially expressed in NK cells from healthy and AML patients

**Figure 1:**
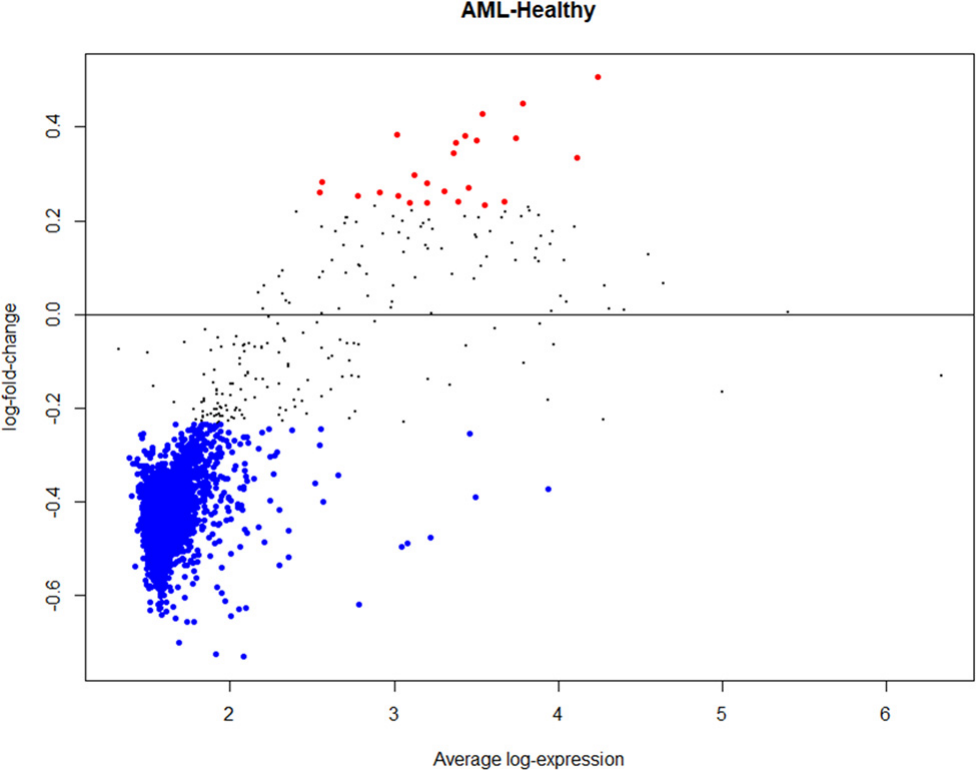
Differentially expressed genes in NK cells isolated from AML patients. In total, 1,834 genes were expressed statistically different between the two groups (adj *p*-value <0.05). Red dots indicate significantly upregulated genes. Blue dots indicate significantly downregulated genes.

**Figure 2:**
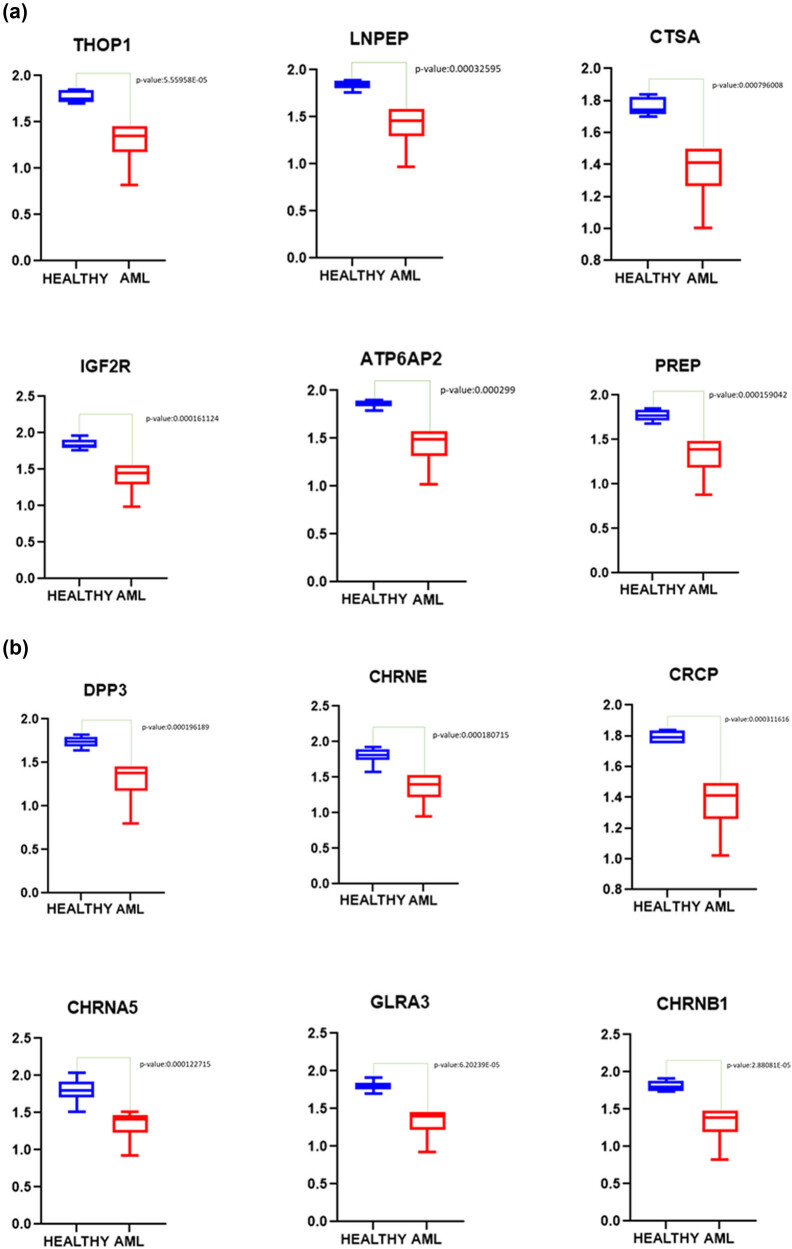
Significantly downregulated genes. Twelve genes were downregulated in the NK cells of AML patients. Six belong to RAS family genes that are significantly downregulated (AML patients) (a). The other six genes belong to Neurotransmitter gene family that are significantly downregulated (AML patients) (b).

**Figure 3:**
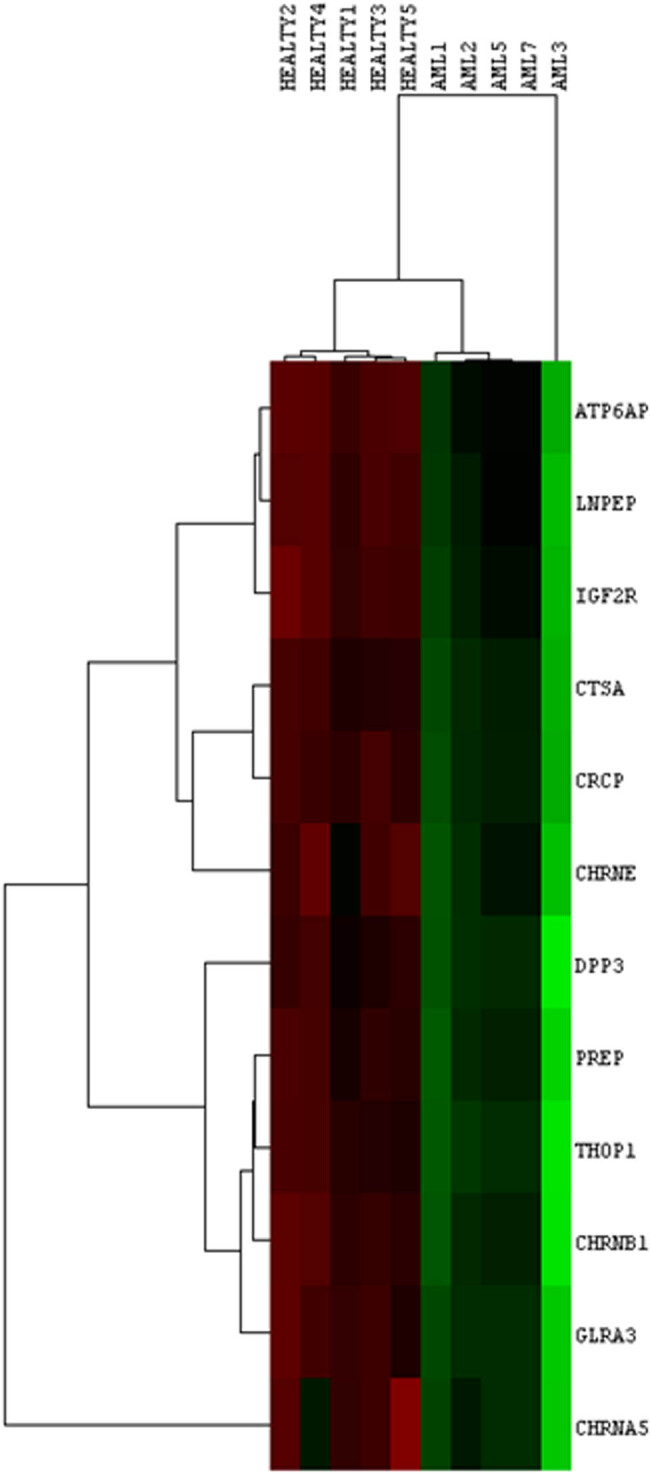
Hierarchical cluster analysis. Clusters of 12 differentially expressed genes with the 5 AML and healthy NK cells. All these genes are downregulated in NK cells isolated from AML patients.

Following normalization of the data, 6 out of 16 samples were discarded due to 0 values for several genes in the samples. To ascertain gene expression alterations in AML patients’ NK cells, whole-genome expression data of NK cells from healthy and AML patient groups were compared. According to the findings, considering the adjusted *p*-values of the genes, 1,834 genes were found to be expressed statistically different between the two groups (adj *p*-value <0.05). All of these genes had log fold change values greater than 0.2 (Figure 1). We determined that six of these genes are connected with the RAS gene family (ATP6AP2, LNPEP, PREP, IGF2R, CTSA, and THOP1) and six are related to neurotransmitter pathways (DPP3, GLRA3, CRCP, CHRNA5, CHRNE, and CHRNB1). Each of these 12 genes was discovered to be downregulated in the NK cells of AML patients [*p*-values were calculated using the limma (version 3.26.8) package in the *R* (version 3.6.3)] (Figure 2a and b). As shown in Figure 3, these genes can distinguish well between healthy and AML patients (Figure 3).

### 3.2 Discovery of enriched gene sets in the NK cells of healthy and AML patients

We used GSEA to discover which gene sets were enriched in the NK cells of healthy and AML patients. According to our findings, gene sets involved in tumor necrosis factor (TNF) superfamily cytokine production, hematopoietic stem cell (HSC) differentiation, scaffold protein binding, protein kinase holoenzyme complex construction, and B-cell proliferation are enriched in NK cells from AML patients (*p*-value 0.05, sizes 71, 61, 29, 27, and 24, respectively). The gene sets enriched in the AML patient group are listed in Table 1. On the other hand, GSEA findings indicate that gene sets associated with cellular response to TGF-β stimuli, nucleotide metabolic processes, and ATP metabolic processes are enriched in the healthy group (*p*-value <0.05, size 18). The most significant gene sets enriched in the healthy group are presented in Table 2.

**Table 1:** Gene sets enriched in NK cells from AML patients

Gene sets	Size	ES	NES	Nom *p*-val	FDR *q*-val	FWER *p*-val	Rank at max
GOBP_TUMOR_NECROSIS_FACTOR_SUPERFAMILY_CYTOKINE_PRODUCTION	71	0.35	2.13	0.006	1	0.229	4,265
GOCC_RESPIRATORY_CHAIN_COMPLEX	75	0.45	2.07	0	1	0.254	2,812
GOCC_PROTEASOME_CORE_COMPLEX	18	0.78	2.01	0	1	0.281	2,147
GOBP_PROTEASOMAL_UBIQUITIN_INDEPENDENT_PROTEIN_CATABOLIC_PROCESS	20	0.69	2.01	0	1	0.281	2,948
GOBP_REGULATION_OF_TRANSCRIPTION_FROM_RNA_POLYMERASE_II_PROMOTER	67	0.38	2	0	1	0.281	2,402
GOBP_ATP_SYNTHESIS_COUPLED_ELECTRON_TRANSPORT	89	0.43	2	0	1	0.281	2,812
GOCC_NADH_DEHYDROGENASE_COMPLEX	48	0.52	1.97	0	1	0.308	2,811
GOCC_RESPIRASOME	88	0.4	1.94	0	1	0.323	3,222
GOBP_ANAPHASE_PROMOTING_COMPLEX_DEPENDENT_CATABOLIC_PROCESS	71	0.35	1.94	0.02	1	0.323	2,290
GOBP_NEGATIVE_REGULATION_OF_TUMOR_NECROSIS_FACTOR_SUPERFAMILY_CY	23	0.35	1.92	0.033	1	0.359	4,265
GOBP_MITOCHONDRIAL_ELECTRON_TRANSPORT_NADH_TO_UBIQUINONE	52	0.49	1.89	0	1	0.418	2,811
GOBP_OXIDATIVE_PHOSPHORYLATION	122	0.36	1.87	0	1	0.435	2,812
GOBP_REGULATION_OF_HEMATOPOIETIC_STEM_CELL_DIFFERENTIATION	61	0.37	1.86	0	1	0.448	4,010
GOBP_REGULATION_OF_CELLULAR_AMINO_ACID_METABOLIC_PROCESS	51	0.4	1.85	0.022	1	0.448	2,290
GOBP_REGULATION_OF_CELLULAR_AMINE_METABOLIC_PROCESS	58	0.36	1.85	0.026	1	0.448	2,290
GOMF_SCAFFOLD_PROTEIN_BINDING	29	0.36	1.85	0	1	0.448	5,778
GOCC_CYCLIN_DEPENDENT_PROTEIN_KINASE_HOLOENZYME_COMPLEX	27	0.39	1.85	0.018	1	0.451	3,005
GOBP_POSITIVE_REGULATION_OF_B_CELL_PROLIFERATION	24	0.47	1.84	0	1	0.451	4,122
GOCC_ENDOPEPTIDASE_COMPLEX	64	0.35	1.84	0	1	0.457	2,363
HP_LIMB_GIRDLE_MUSCLE_ATROPHY	18	0.46	1.83	0.01	1	0.49	2,462

**Table 2:** Gene sets enriched in NK cells from healthy donors

Gene sets	Size	ES	NES	Nom *p*-Val	FDR *q*-val	FWER *p*-Val	Rank at max
HP_MOTOR_AXONAL_NEUROPATHY	20	−0.45	−1.73	0.01	1	0.695	3,963
HP_3_METHYLGLUTACONIC_ACIDURIA	16	−0.43	−1.7	0.004	1	0.8	4,503
HP_ABNORMAL_CIRCULATING_FATTY_ACID_ANION_CONCENTRATION	15	−0.54	−1.68	0	1	0.832	2,057
GOMF_LIGASE_ACTIVITY_FORMING_CARBON_OXYGEN_BONDS	15	−0.53	−1.62	0.016	1	0.938	3,341
GOBP_POSITIVE_REGULATION_OF_CELLULAR_RESPONSE_TO_TRANSFORMING_GROWTH_FACTOR_BETA_STIMULUS	18	−0.39	−1.58	0.048	1	0.955	4,843
GOBP_TETRAPYRROLE_BIOSYNTHETIC_PROCESS	22	−0.38	−1.57	0.045	1	0.959	2,901
HP_ABNORMAL_URINE_CARBOXYLIC_ACID_LEVEL	30	−0.38	−1.52	0.041	1	0.977	3,589
GOMF_SODIUM_ION_TRANSMEMBRANE_TRANSPORTER_ACTIVITY	28	−0.33	−1.51	0.087	1	0.989	1,979
GOBP_PIGMENT_BIOSYNTHETIC_PROCESS	35	−0.29	−1.49	0.109	1	0.989	2,901
GOBP_REGULATION_OF_NUCLEOTIDE_BIOSYNTHETIC_PROCESS	16	−0.37	−1.49	0.094	1	0.989	1,704
GOMF_LIGAND_GATED_CATION_CHANNEL_ACTIVITY	17	−0.44	−1.48	0.123	1	0.991	3,292
GOMF_CATALYTIC_ACTIVITY_ACTING_ON_A_TRNA	75	−0.34	−1.47	0.135	1	0.991	4,263
GOMF_LIGAND_GATED_ION_CHANNEL_ACTIVITY	19	−0.42	−1.47	0.134	1	0.993	3,292
HP_TYPE_1_MUSCLE_FIBER_PREDOMINANCE	19	−0.36	−1.46	0.138	1	0.993	2,667
GOBP_POSITIVE_REGULATION_OF_NUCLEOTIDE_METABOLIC_PROCESS	18	−0.35	−1.46	0.102	1	0.993	1,357
GOBP_POSITIVE_REGULATION_OF_ATP_METABOLIC_PROCESS	18	−0.35	−1.46	0.102	1	0.993	1,357
HP_ABNORMAL_SPERM_MOTILITY	17	−0.39	−1.45	0.061	1	0.993	2,822
GOBP_GLUTAMATE_METABOLIC_PROCESS	16	−0.41	−1.45	0.09	1	0.993	4,643
HP_BILATERAL_TONIC_CLONIC_SEIZURE_WITH_GENERALIZED_ONSET	23	−0.34	−1.45	0.067	1	0.994	3,068
GOMF_TRNA_METHYLTRANSFERASE_ACTIVITY	25	−0.35	−1.43	0.172	1	0.994	4,166

**Figure 4:**
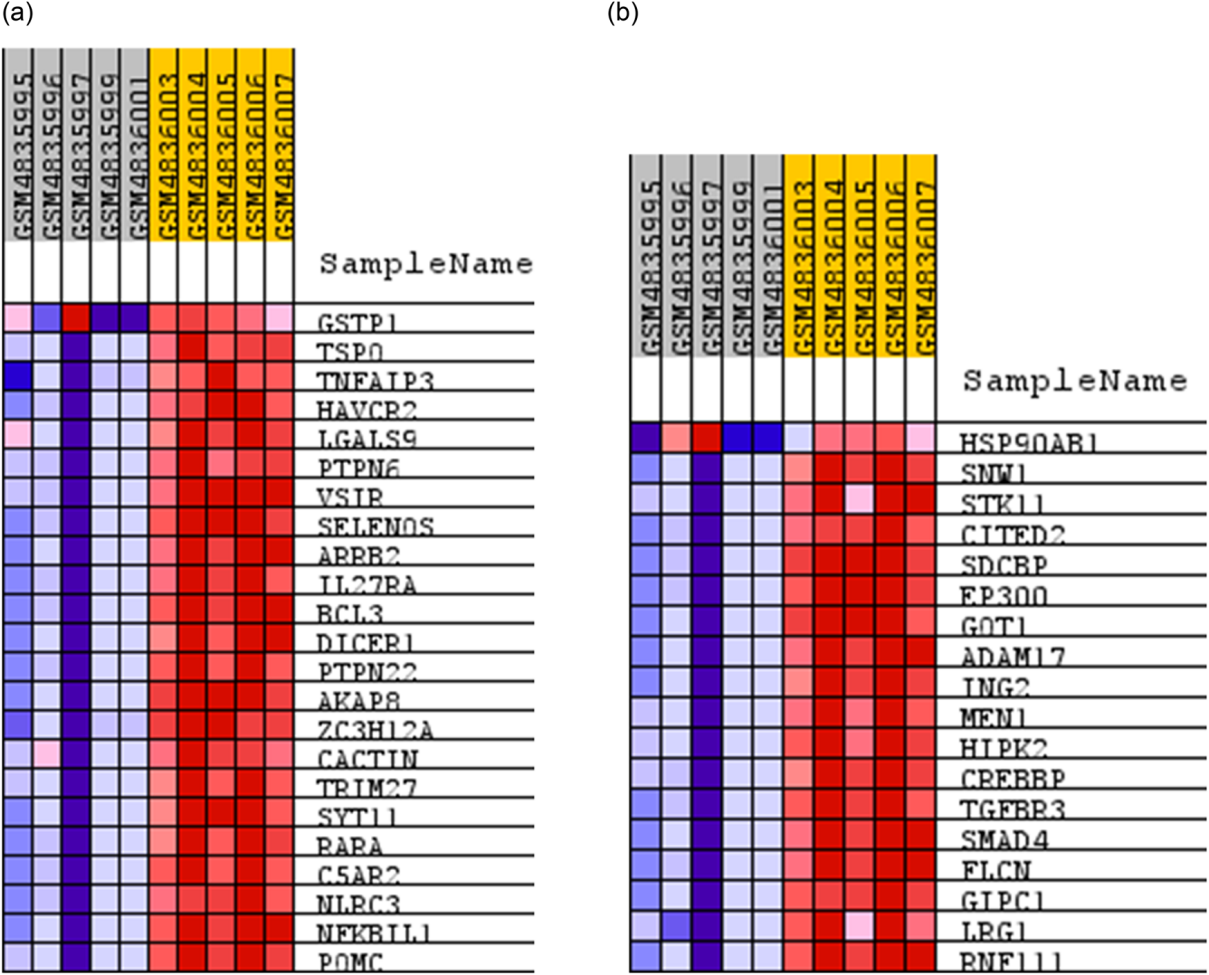
GESA of AML and healthy NK cells. Gene sets enriched in AML NK cells with an FDR *q*-value of lower than 0.25 included TNF superfamily cytokine production (a). Gene sets enriched in healthy NK cells with an FDR *q*-value of lower than 0.25 included TGF-β stimuli (b).

To determine molecular mechanisms underlying differential response of NK cells of AML and healthy donors, we analyzed GSEA results. Several gene sets were significantly enriched among NK cells of AML and healthy donors (FDR *q*-value <0.25). But especially, gene sets enriched in AML NK cells with an FDR *q*-value of lower than 0.25 included TNF superfamily cytokine production; while gene sets, such as stimuli of TGF-β, were enriched in healthy NK cells (Figure 4a and b).

### 3.3 Seven distinct groups from the most differentially expressed genes

Clustering analysis of functional annotations for the most differentially expressed 100 genes revealed seven distinct groupings. According to their enrichment scores, the first cluster (ES: 2.05) contains the majority of genes involved in neurotransmitter-related pathways, the second cluster (ES: 1.93) contains genes associated with RAS and RAS-related pathways, and the third cluster (ES: 0.83) contains genes associated with zinc- and metal-binding pathways (Table 3). All seven clusters regarding functional annotation analysis are presented in Table S1.

**Table 3:** Functional annotation analysis of first 100 differentially expressed genes

Annotation cluster 1	Pathways enrichment score: 2.05	*p-*Value	Benjamini
INTERPRO	Neurotransmitter-gated ion-channel. conserved site	8.80 × 10^−4^	6.00 × 10^−2^
INTERPRO	Neurotransmitter-gated ion-channel transmembrane domain	1.00 × 10^−3^	6.00 × 10^−2^
INTERPRO	Neurotransmitter-gated ion-channel	1.00 × 10^−3^	6.00 × 10^−2^
INTERPRO	Neurotransmitter-gated ion-channel ligand binding	1.00 × 10^−3^	6.00 × 10^−2^
INTERPRO	Nicotinic acetylcholine receptor	2.40 × 10^−3^	1.20 × 10^−1^
UP_KEYWORDS	Synapse	2.80 × 10^−3^	2.20 × 10^−1^
UP_KEYWORDS	Ion channel	2.90 × 10^−3^	2.20 × 10^−1^
UP_KEYWORDS	Ligand-gated ion channel	3.90 × 10^−3^	2.20 × 10^−1^
GOTERM_MF_DIRECT	Acetylcholine receptor activity	4.20 × 10^−3^	3.50 × 10^−1^
GOTERM_MF_DIRECT	Acetylcholine-activated cation-selective channel activity	4.20 × 10^−3^	3.50 × 10^−1^
INTERPRO	Nicotinic acetylcholine-gated receptor, transmembrane domain	4.30 × 10^−3^	1.70 × 10^−1^
GOTERM_CC_DIRECT	Acetylcholine-gated channel complex	4.30 × 10^−3^	6.10 × 10^−10^
GOTERM_MF_DIRECT	Acetylcholine binding	5.30 × 10^−3^	3.50 × 10^−1^
UP KEYWORDS	Postsynaptic cell membrane	5.50 × 10^−3^	2.30 × 10^−1^
GOTERM_BP_DIRECT	Neuromuscular synaptic transmission	5.50 × 10^−3^	1.00 × 10^00^
GOTERM MF_DIRECT	Ligand-gated ion channel activity	1.00 × 10^−2^	4.90 × 10^−1^
GOTERM_BP_DIRECT	Synaptic transmission, cholinergic	1.10 × 10^−2^	1.00 × 10^00^

Annotation cluster 2	Pathways enrichment score: 1.93	*p-*value	Benjamini
KEGG_PATHWAY	RAS	4.60 × 10^−6^	3.60 × 10^−4^
GOTERM_BP_DIRECT	Proteolysis	2.00 × 10^−2^	1.00 × 10^00^
GOTERM_MF_DIRECT RT	Metallopeptidase activity	4.60 × 10^−2^	1.00 × 10^00^
UP_KEYWORDS	Protease	6.30 × 10^−2^	8.00 × 10^−1^

Annotation cluster 3	Pathways enrichment score: 0.83	*p-*value	Benjamini
UP_KEYWORDS	Zinc	4.50 × 10^−1^	1.00 × 10^00^
GOTERM_MF DIRECT	Zinc ion binding	2.20 × 10^−1^	1.00 × 10^00^
UP_KEYWORDS	Metal binding	3.60 × 10^−1^	1.00 × 10^00^
INTERPRO	Zinc finger C_2_H_2_-type/integrase DNA binding domain	7.90 × 10^−1^	1.00 × 10^00^
SMART	ZnF C_2_H_2_	7.90 × 10^−1^	1.00 × 10^00^
INTERPRO	Zinc finger, C_2_H_2_-like	8.20 × 10^−1^	1.00 × 10^00^
UP_KEYWORDS	Zinc finger	8.40 × 10^−1^	1.00 × 10^00^
UP_SEQ_FEATURE	Metal ion-binding site: zinc; catalytic	0.092	1
INTERPRO	Zinc finger, C_2_H_2_	8.40 × 10^−1^	1.00 × 10^00^

## 4 Discussion

The bioinformatic analyses undertaken in this work focused on two critical pathways and associated genes to identify NK-cell dysfunction in AML patients. In this study, we showed that the downregulation of some critical genes related to the RAS gene family and neurotransmitter-associated pathways might be responsible for the inappropriate and dysfunctional NK cells in AML patients. Novel pathways and genes related to RAS and neurotransmitters, which play a significant role in NK biology and proper function in patients with AML, were also focused and directed in this work.

In addition to immunological responses and inflammation, the RAS is made up of a variety of substrates and enzymes that may work together or independently [13]. The identification of local RAS in the heart, brain, pancreas, lymphatic, and adipose tissue was a real advance in the area. Local RAS may work alone or in conjunction with circulating RAS. A functioning intracellular RAS has also been found, showing angiotensin II’s (AngII) pro-inflammatory, proliferative, and fibrotic properties [14,15]. It regulates the generation of free radicals and the cellular synthesis of cytokines, chemokines, and transcription factors and modulates cell proliferation and differentiation via these mechanisms. RAS and its primary effector molecule, AngII, are implicated in inflammation and immunity [16]. AngII enhances their chemotaxis, perhaps amplifying inflammation [17]. It is yet unknown if AngII has a direct influence on the function and cellular location of certain immune cells in humans. T and NK cells have been shown to be capable of generating and transporting AngII to inflammatory areas. AngII therapies enhanced the proliferation of T and NK cells activated by mitogens and anti-CD3. This research implies that NK and T cells are capable of producing functional renin and angiotensin-converting enzyme (ACE) activity.

According to the findings of the current study, ATP6AP2, LNPEP, PREP, IGF2R, CTSA, and THOP1 genes belonging to the RAS gene family were discovered to be downregulated in the NK cells of AML patients.

As supported, ATP6AP2 is involved in the regulation of organellar, cellular, systemic homeostasis, and activating this receptor is essential for the conversion of angiotensinogen to angiotensin [18]. ATP6AP2 is upregulated in NK cells compared to CD8^+^ T cells [19]. LNPEP is capable of degrading peptide hormones, such as oxytocin, vasopressin, and angiotensin III (AngIII) [20]. AngIII, like AngII, increases bvasopressin release and promotes the production of pro-inflammatory regulators [21].

The IGF2R activates proton rechanneling into the mitochondrial intermembrane area, allowing for persistent OXPHOS [22,23]. L-IGF2 increases DNA methylation by activating GSK3. This sequestered v-ATPase assembly prevents protons from reaching lysosomes and redirects them to mitochondria [24].

CTSA is required for proper lysosomal routing, stability, and activation of β-galactosidase and alpha-neuraminidase. It also inactivates bioactive peptides. Cytotoxic chemicals are stored in secretory lysosomes, an exocytic organelle present in NK cells [25]. Thus, changes in the expression of genes that control or affect lysosomal activity may affect the cytotoxicity of NK cells, as well.

THOP1 assists with present MHC-I antigens [26]. It is involved in short-term neuropeptide metabolism. Cytoplasmic peptides degrade amyloid-β precursor protein fragments. Peptide transport disruption appears to impact NK cells [26]. NK cells interact with dendritic cells to destroy target cells and modulate innate and adaptive immunity [27]. Any MHC1 presentation issue may impact NK-cell function. Based on our findings, DPP3, GLRA3, CRCP, CHRNA5, CHRNE, and CHRNB1 genes that are involved in neurotransmitter pathways were found to be downregulated in the NK cells of AML patients as well.

Dipeptidyl peptidase (DPP4), a member of the dipeptidyl peptidase family, is present in trace concentrations on newly isolated human NK cells and is increased in a small subpopulation in response to interleukin (IL)-2 stimulation [28]. In addition, it was shown that inhibiting DPP4 inhibits DNA synthesis and cell cycle progression in NK cells. Interestingly, in a model of lung metastasis, NK cytolytic ability against tumor cells was decreased in DPP4-deficient mice [29]. Glycine (Gly) is a neurotransmitter that acts mostly in the central nervous system’s caudal region. Flow cytometry has shown the presence of GlyR in human NK cells. This receptor may influence NK-cell activity in AML patients [30]. It was shown that calcitonin gene-related peptide, or CGRP, has a dose-dependent effect on the NK activity of spleen cells in mice [31]. So, CGRP may be involved in regulating NK function. It has also been established that CGRP may influence the activity of macrophages in sensory neuron nerve terminals during the onset and maintenance of inflammation. CHRNA5, CHRNB1, and CHRNE from the cholinergic receptor nicotinic gene family were found to be downregulated in the NK cells of AML patients in our study. Both adaptive and innate immune cells are controlled by the cholinergic system, which mediates their mobilization, differentiation, and antigen presentation. NK activation with cytokines increases mRNA and protein levels of the nicotinic receptor 7 have been demonstrated [32]. It has been suggested that the expression level of nicotinic receptors is critical to the effective activity of NK cells. NK cells perform their cytotoxic function by secreting cytokines, which cause malignant or infected cells to rupture and death.

Aside from identifying specific genes whose expression differs between healthy and AML NK cells, the second important component of our work was the identification of critical pathways and gene sets associated with NK dysfunction. As revealed by our findings, TNF superfamily cytokine production, HSC differentiation, scaffold protein binding, kinase holoenzyme complex construction, and B-cell proliferation are enriched in NK cells from patients with AML. Gene sets related with cellular response to TGF-β stimuli, nucleotide metabolic activities, and ATP metabolic processes, on the other hand, were shown to be enriched in the healthy group, according to GSEA results.

Tumor development and metastasis are slowed or stopped entirely when interferon and TNF-α are released or membrane bound by NK cells. NK cells are the primary immune effectors that play a role in this process. It was discovered that, in addition to TNF alpha, NK cells relied on additional members of the TNF family. It has been revealed that the TNF superfamily member TRAIL plays a significant role in tumor defense by NK cells or CD8^+^ T lymphocytes [33]. It has been shown that TNF receptors are involved in the formation of lymphokine-activated killer cell activity and the multiplication of NK cells [34].

On the other hand, TNF production is elevated in the presence of the angiotensin subtype-1 receptor, which has been demonstrated to affect inflammatory processes by increasing the signal transduction and activator of transcription proteins 3 (STAT3) signaling [35]. In addition, studies suggest that increased AT2R expression may play a role in the observed decrease in the inflammatory pathway activation through decreased TNF-α production and STAT3 signaling. AT2R expression and/or activation are the possible therapeutic target for the control of inflammation [36]. Meanwhile, TNF superfamily members have also been demonstrated to have an impact on the production and function of a variety of neurotransmitters [37,38,39,40].

Cytokine stimuli activate HSCs. Self-renewing multipotent HSCs commit to common lymphoid progenitor status in response to certain stimuli. Progenitors of NK cells are generated by non-hematopoietic cells, such as mesenchymal stem cells, that release IL-7 or IL-15, which play a variety of functions in programming CLPs (NK-cell progenitors). It is possible to functionally develop immature NK cells into well-defined subsets of adult NK cells using chimeric antigen receptor cells. The sinusoidal blood channels carry mature NK cells to the secondary lymphoid organs. The formation, development, and function of NK cells will be affected if this process is disrupted. In addition, ACE and its major enzymatic product, Ang II, are recognized as critical determinants of angiogenesis, inflammation, tumor progression, and hematopoiesis [41]. Studies also identified ACE as a marker of both adult and embryonic human hematopoietic stem/progenitor cells [42]. On the other hand, HSC niche is regulated in part by the neurological system and neurotransmitters [43].

Scaffolding proteins play role in stabilizing the structure of other enriched gene sets inside the NK of AML patients. Multiple protein–protein interaction modules are common among the many scaffolding proteins that exist in nature [44]. A number of critical signaling pathways rely on scaffold proteins, which are proteins that interact with numerous elements of a route and bind them together form complexes [45]. It is possible that the alterations in the gene sets that regulate the binding of the scaffold proteins may contribute to the degradation of NK-cell activities by disrupting numerous essential signal pathways, such as RAS and neurotransmitter.

Furthermore, gene sets involved in B-cell proliferation were found to be enriched in the AML group. Circulating RAS and local paracrine–autocrine–intracrine tissue-based RAS participates in numerous pathobiological events [46]. Multiple components of the RAS signaling cascade influence inflammatory cell phenotype and function with unpredictable and context-specific effects on innate and adaptive immunities [13].

However, in NK cells from healthy donors, TGF-β stimulates an increased response. Anti-inflammatory cytokines like TGF-β may be produced in response to the pro-inflammatory cellular and molecular environment. Activated lymphocytes, macrophages, and dendritic cells create these anti-inflammatory cytokines, which may reduce the immune cell activity. When it comes to preventing the spread of infectious diseases, the immune system is the most important weapon. It is founded that tumor cell death produced by NK cells was reduced *in vitro* by metastasis-associated lung macrophages, which were shown to be membrane bound and reliant on the growth factor TGF-β [47]. This process is important for the prompt arrest of NK cells. TGF-β synthesis is boosted by RAS components. RAS and TGF-β have crucial clinical consequences for each other’s relationship [13]. Reduction in RAS inhibitory effects is directly linked to the reduction of TGF-β production in both experimental and clinical renal disorders [48].

NK cells of normal individuals are also rich in two additional gene sets, nucleotide metabolic activities and ATP metabolic processes, both of which seem to be linked to a wide range of critical cell processes.

In addition, we determined that the nucleotide and metabolic processes in healthy donors’ NK cells are favorably regulated. The importance of immunometabolism in the control of NK-cell activity is becoming more widely recognized. An imbalanced metabolic profile and defective mitochondria in NK cells resulted in an inability to destroy them. As a result, NK-cell metabolic pathways are altered, resulting in tumor-promoting settings with NK-cell malfunction. Furthermore, we identified seven clusters of genes that differed significantly between healthy and AML NK cells. According to our findings, the best clusters were linked to neurotransmitter pathways, RAS, and zinc-related pathways. There is a connection with the neurological and immune systems because each of the systems expresses receptors for neurotransmitters. Because autocrine and paracrine systems allow immune cells to produce and release neurotransmitters themselves, neurotransmitter-mediated pathways are used. The neurological system and several neurotransmitter-related pathways have a significant impact on NK-cell function. The immune system may be modulated by zinc, a vital trace element. Zinc supplementation increases perforin expression, which promotes NK-cell function. The activity of NK cells was also raised by zinc supplementation, which also elevated IL-2. Zinc deficiency, on the other hand, decreased NK-cell activity and dampened the IL-2 medication’s boosting impact on NK cells [49,50].

The RAS regulates immune and cancer cells in fundamental ways [48]. All of the many RAS molecules play an important role in controlling inflammation and other immune responses [51]. Most innate and adaptive immune cells exhibit components of the RAS pathway. RAS signaling seems to influence the activity of numerous immune cells in both *in vitro* and *in vivo*.

The *in silico* design may be considered the limitation of our study. However, based on the results of this present study, the expression of RAS and neurotransmitter genes in NK cells from AML patients could influence the (patho)biological course of anti-tumor NK responses.

The immune and neurological systems are dependent on one another for their fine tuning and functioning, and they work together to preserve physiological homeostasis and prevent infection as well as the development of cancer, respectively. According to our research, neurotransmitters have been proven to have a significant influence on the biology and functioning of NK cells. On the other hand, it has been shown that the RAS gene family plays a crucial role in the development of NK cells. Important genes belonging to these two gene families were highlighted in this work for the first time, and the interaction between RAS and neurotransmitters was investigated. In the context of these data, the next step in research planning should be the experiments in the laboratory and in the clinic for *in vitro* and clinical validation.

## Supplementary Material

Supplementary Table
