# Supplementary material for: NK-cell dysfunction of acute myeloid leukemia in relation to the renin–angiotensin system and neurotransmitter genes
Source: Open Med (Wars). 2022 Sep 20;17(1):1495–506. doi: 10.1515/med-2022-0551 (PMC9490854; doi:10.1515/med-2022-0551)
Supplement: Supplementary Table [file med-2022-0551-sm.pdf]

# Supplementary material

**Table S1:** All clusters regarding functional annotation analysis are presented

| Annotation Cluster 1 | Pathways Enrichment Score: 2.05 | *P* value | Benjamini |
|---|---|---|---|
| INTERPRO | Neurotransmitter-gated ion-channel. conserved site | $8.80 \times 10^{-4}$ | $6.00 \times 10^{-2}$ |
| INTERPRO | Neurotransmitter-gated ion-channel transmembrane domain | $1.00 \times 10^{-3}$ | $6.00 \times 10^{-2}$ |
| INTERPRO | Neurotransmitter-gated ion-channel | $1.00 \times 10^{-3}$ | $6.00 \times 10^{-2}$ |
| INTERPRO | Neurotransmitter-gated ion-channel ligand binding | $1.00 \times 10^{-3}$ | $6.00 \times 10^{-2}$ |
| INTERPRO | Nicotinic acetylcholine receptor | $2.40 \times 10^{-3}$ | $1.20 \times 10^{-1}$ |
| UP_KEYWORDS | Synapse | $2.80 \times 10^{-3}$ | $2.20 \times 10^{-1}$ |
| UP_KEYWORDS | Ion channel | $2.90 \times 10^{-3}$ | $2.20 \times 10^{-1}$ |
| UP_KEYWORDS | Ligand-gated ion channel | $3.90 \times 10^{-3}$ | $2.20 \times 10^{-1}$ |
| GOTERM_MF_DIRECT | acetylcholine receptor activity | $4.20 \times 10^{-3}$ | $3.50 \times 10^{-1}$ |
| GOTERM_MF_DIRECT | acetylcholine-activated cation-selective channel activity | $4.20 \times 10^{-3}$ | $3.50 \times 10^{-1}$ |
| INTERPRO | Nicotinic acetylcholine-gated receptor, transmembrane domain | $4.30 \times 10^{-3}$ | $1.70 \times 10^{-1}$ |
| GOTERM_CC_DIRECT | acetylcholine-gated channel complex | $4.30 \times 10^{-3}$ | $6.10 \times 10^{-10}$ |
| GOTERM_MF_DIRECT | acetylcholine binding | $5.30 \times 10^{-3}$ | $3.50 \times 10^{-1}$ |
| UP KEYWORDS | Postsynaptic cell membrane | $5.50 \times 10^{-3}$ | $2.30 \times 10^{-1}$ |
| GOTERM_BP_DIRECT | neuromuscular synaptic transmission | $5.50 \times 10^{-3}$ | $1.00 \times 10^{00}$ |
| GOTERM MF_DIRECT | ligand-gated ion channel activity | $1.00 \times 10^{-2}$ | $4.90 \times 10^{-1}$ |
| GOTERM_BP_DIRECT | synaptic transmission, cholinergic | $1.10 \times 10^{-2}$ | $1.00 \times 10^{00}$ |
| **Annotation Cluster 2** | **Pathways Enrichment Score: 1.93** | ***P* value** | **Benjamini** |
| KEGG_PATHWAY | Renin-angiotensin system | $4.60 \times 10^{-6}$ | $3.60 \times 10^{-4}$ |
| GOTERM_BP_DIRECT | proteolysis | $2.00 \times 10^{-2}$ | $1.00 \times 10^{00}$ |
| GOTERM_MF_DIRECT RT | metallopeptidase activity | $4.60 \times 10^{-2}$ | $1.00 \times 10^{00}$ |
| UP_KEYWORDS | Protease | $6.30 \times 10^{-2}$ | $8.00 \times 10^{-1}$ |
| **Annotation Cluster 3** | **Pathways Enrichment Score: 0.83** | ***P* value** | **Benjamini** |
| UP_KEYWORDS | Zinc | $4.50 \times 10^{-1}$ | $1.00 \times 10^{00}$ |
| GOTERM_MF DIRECT | zinc ion binding | $2.20 \times 10^{-1}$ | $1.00 \times 10^{00}$ |
| UP_KEYWORDS | Metal-binding | $3.60 \times 10^{-1}$ | $1.00 \times 10^{00}$ |
| INTERPRO | Zinc finger C2H2-type/integrase DNA binding domain | $7.90 \times 10^{-1}$ | $1.00 \times 10^{00}$ |
| SMART | ZnF C2H2 | $7.90 \times 10^{-1}$ | $1.00 \times 10^{00}$ |
| INTERPRO | Zinc finger, C2H2-like | $8.20 \times 10^{-1}$ | $1.00 \times 10^{00}$ |
| UP_KEYWORDS | Zinc-finger | $8.40 \times 10^{-1}$ | $1.00 \times 10^{00}$ |
| UP_SEQ_FEATURE | metal ion-binding site: Zinc; catalytic | 0.092 | 1 |
| INTERPRO | Zinc finger, C2H2 | $8.40 \times 10^{-1}$ | $1.00 \times 10^{00}$ |

(*Continued*)

**Table S1:** *Continued*

| **Annotation Cluster 3** | **Pathways Enrichment Score: 0.83** | ***P* value** | **Benjamini** |
|---|---|---|---|
| Annotation Cluster 4 | Pathways Enrichment Score: 0.59 | *P* value | Benjamini |
| GOTERM_CC DIRECT | postsynaptic membrane | $1.10 \times 10^{-2}$ | $7.80 \times 10^{-1}$ |
| UP_KEYWORDS | Ion transport | $1.30 \times 10^{-2}$ | $3.90 \times 10^{-1}$ |
| GOTERM_BP_DIRECT | Ion transport | $1.70 \times 10^{-2}$ | $1.00 \times 10^{00}$ |
| GOTERM_BP DIRECT | cation transmembrane transport | $1.80 \times 10^{-2}$ | $1.00 \times 10^{00}$ |
| UP_KEYWORDS | Transport | $2.20 \times 10^{-2}$ | $4.20 \times 10^{-1}$ |
| GOTERM_CC DIRECT | cell junction | $4.10 \times 10^{-2}$ | $1.00 \times 10^{00}$ |
| UP_KEYWORDS | cell junction | $5.00 \times 10^{-2}$ | $7.10 \times 10^{-1}$ |
| KEGG PATHWAY | Neuroactive ligand-receptor interaction | $1.70 \times 10^{-1}$ | $1.00 \times 10^{00}$ |
| UP_KEYWORDS | Receptor | $2.00 \times 10^{-1}$ | $1.00 \times 10^{00}$ |
| GOTERM_BP_DIRECT | chemical synaptic transmission | $2.80 \times 10^{-1}$ | $1.00 \times 10^{00}$ |

| **Annotation Cluster 5** | **Pathways Enrichment Score: 0.55** | ***P* value** | **Benjamini** |
|---|---|---|---|
| GOTERM_CC_DIRECT | integral component of plasma membrane | $7.00 \times 10^{-1}$ | $1.00 \times 10^{00}$ |
| UP_KEYWORDS | Ion channel | $2.90 \times 10^{-3}$ | $2.20 \times 10^{-1}$ |
| UP_KEYWORDS | Ion channel | $1.30 \times 10^{-2}$ | $3.90 \times 10^{-1}$ |
| UP_KEYWORDS | Chloride channel | $1.70 \times 10^{-2}$ | $4.20 \times 10^{-1}$ |
| UP_KEYWORDS | Chloride | $3.40 \times 10^{-2}$ | $5.20 \times 10^{-1}$ |
| GOTERM_BP_DIRECT | chloride transmembrane transport | $6.00 \times 10^{-2}$ | $1.00 \times 10^{00}$ |
| UP_SEQ_FEATURE | metal ion-binding site: Zinc; catalytic | $9.20 \times 10^{-2}$ | $1.00 \times 10^{00}$ |
| UP_KEYWORDS | Metalloprotease | $1.20 \times 10^{-1}$ | $1.00 \times 10^{00}$ |
| UP_KEYWORDS | Hydrolase | $3.40 \times 10^{-1}$ | $1.00 \times 10^{00}$ |
| GOTERM_BP_DIRECT | positive regulation of GTPase activity | $2.20 \times 10^{-1}$ | $1.00 \times 10^{00}$ |

| **Annotation Cluster 6** | **Pathways Enrichment Score: 0.37** | ***P* value** | **Benjamini** |
|---|---|---|---|
| UP_SEQ FEATURE | domain:PH | $2.50 \times 10^{-1}$ | $1.00 \times 10^{00}$ |
| INTERPRO | Pleckstrin homology-like domain | $2.50 \times 10^{-1}$ | $1.00 \times 10^{00}$ |
| SMART | PH | $2.60 \times 10^{-1}$ | $1.00 \times 10^{00}$ |
| UP_SEQ_FEATURE | transmembrane region | $1.20 \times 10^{-1}$ | $1.00 \times 10^{00}$ |
| UP_KEYWORDS | Transmembrane helix | $2.80 \times 10^{-1}$ | $1.00 \times 10^{00}$ |
| UP_KEYWORDS | Membrane | $1.80 \times 10^{-1}$ | $1.00 \times 10^{00}$ |
| UP_SEQ_FEATURE | topological domain:Cytoplasmic | $2.80 \times 10^{-1}$ | $1.00 \times 10^{00}$ |
| UP_KEYWORDS | Transmembrane | $2.80 \times 10^{-1}$ | $1.00 \times 10^{00}$ |
| UP_SEQ_FEATURE | topological domain: Extracellular | $3.00 \times 10^{-1}$ | $1.00 \times 10^{00}$ |
| UP_SEQ_FEATURE | glycosylation site: N-linked (GlcNAc...) | $3.00 \times 10^{-1}$ | $1.00 \times 10^{00}$ |
| GOTERM_CC DIRECT | integral component of membrane | $4.70 \times 10^{-1}$ | $1.00 \times 10^{00}$ |
| UP_KEYWORDS | Glycoprotein | $5.30 \times 10^{-1}$ | $1.00 \times 10^{00}$ |

| **Annotation Cluster 7** | **Pathways Enrichment Score: 0.11** | ***P* value** | **Benjamini** |
|---|---|---|---|
| UP_KEYWORDS | Calmodulin-binding | $2.20 \times 10^{-2}$ | $4.20 \times 10^{-1}$ |

(*Continued*)

**Table S1:** *Continued*

| Annotation Cluster 7 | Pathways Enrichment Score: 0.11 | *P* value | Benjamini |
|---|---|---|---|
| GOTERM_MF DIRECT | calmodulin binding | $4.50 \times 10^{-2}$ | $1.00 \times 10^{00}$ |
| INTERPRO | Serine/threonine-protein kinase, active site | $3.70 \times 10^{-1}$ | $1.00 \times 10^{00}$ |
| UP_SEQ_FEATURE | binding site:ATP | $3.80 \times 10^{-1}$ | $1.00 \times 10^{00}$ |
| SMART | S TKC | $4.00 \times 10^{-1}$ | $1.00 \times 10^{00}$ |
| UP_KEYWORDS | Serine/threonine-protein kinase | $4.60 \times 10^{-1}$ | $1.00 \times 10^{00}$ |
| INTERPRO | Protein kinase, ATP binding site | $4.60 \times 10^{-1}$ | $1.00 \times 10^{00}$ |
| UP_SEQ_FEATURE | nucleotide phosphate-binding region: ATP | $5.50 \times 10^{-1}$ | $1.00 \times 10^{00}$ |
| UP_KEYWORDS | Methylation | $5.60 \times 10^{-1}$ | $1.00 \times 10^{00}$ |
| UP_SEQ_FEATURE | domain:Protein kinase | $5.60 \times 10^{-1}$ | $1.00 \times 10^{00}$ |
| GOTERM_BP_DIRECT | protein phosphorylation | $5.90 \times 10^{-1}$ | $1.00 \times 10^{00}$ |
| INTERPRO | Protein kinase, catalytic domain | $6.00 \times 10^{-1}$ | $1.00 \times 10^{00}$ |
| INTERPRO | Protein kinase-like domain | $6.40 \times 10^{-1}$ | $1.00 \times 10^{00}$ |
| UP_KEYWORDS | ATP-binding | $6.50 \times 10^{-1}$ | $1.00 \times 10^{00}$ |
| UP_KEYWORDS | Nucleotide-binding | $7.20 \times 10^{-1}$ | $1.00 \times 10^{00}$ |
| UP_SEQ_FEATURE | active site:Proton acceptor | $7.50 \times 10^{-1}$ | $1.00 \times 10^{00}$ |
| GOTERM MF DIRECT | ATP-binding | $7.60 \times 10^{-1}$ | $1.00 \times 10^{00}$ |
| UP_KEYWORDS | Kinase | $7.90 \times 10^{-1}$ | $1.00 \times 10^{00}$ |
| UP_SEQ_FEATURE | glycosylation site:N-linked (GlcNAc...) | $3.00 \times 10^{-1}$ | $1.00 \times 10^{00}$ |
| UP_KEYWORDS | Signal | $3.70 \times 10^{-1}$ | $1.00 \times 10^{00}$ |
| UP_SEQ_FEATURE | signal peptide | $4.50 \times 10^{-1}$ | $1.00 \times 10^{00}$ |
| UP_SEQ_FEATURE | disulfide bond | $4.80 \times 10^{-1}$ | $1.00 \times 10^{00}$ |
| UP_KEYWORDS | Disulfide bond | $6.10 \times 10^{-1}$ | $1.00 \times 10^{00}$ |
| GOTERM_CC DIRECT | extracellular space | $6.60 \times 10^{-1}$ | $1.00 \times 10^{00}$ |
| GOTERM_CC_DIRECT | extracellular region | $8.00 \times 10^{-1}$ | $1.00 \times 10^{00}$ |
| UP_KEYWORDS | Secreted | $9.00 \times 10^{-1}$ | $1.00 \times 10^{00}$ |
